# Supplementary material for: Robust prediction of drug combination side effects in realistic settings
Source: PLoS Comput Biol. 2026 Oct 5;22(10):e1013619. doi: 10.1371/journal.pcbi.1013619 (PMC13657403; doi:10.1371/journal.pcbi.1013619)
Supplement: Supplementary file 1 [file pcbi.1013619.s001.pdf]

Supplementary Material to

Robust prediction of drug combination side effects in realistic settings

Rubén Jiménez[1], Alberto Paccanaro[1,2]

[1] Escola de Matemática Aplicada, Fundação Getúlio Vargas, Rio de Janeiro, Brazil
[2] Department of Computer Science, Centre for Systems and Synthetic Biology, Royal Holloway University of London, Egham, UK

Correspondence should be addressed to A.P. (alberto.paccanaro@rhul.ac.uk)

Linear combination of drug signatures

In this section, we show our initial approach of learning the combination signature through a simple linear combination of the signatures of individual drugs. The starting point is the architecture shown in Fig A. We begin with the signatures of the individual drugs, concatenate them, and connect them to a single layer that learns weights for a linear combination. This combination produces the signature for the drug pair. The overall architecture resembles that of DCSE, except that it does not include an MLP to learn the combination signature. This approach – termed DCSE-linear – was evaluated using the same training and testing sets from the Zitnik et al. experimental setup.

While the results obtained for DCSE-linear suggests that learning a signature for drug combinations is promising, the results in Table A indicate this is a highly nonlinear task. The substantial improvement of all the non-linear methods over DCSE-linear demonstrates that a linear model cannot effectively capture the complexity involved in combining drug signatures.

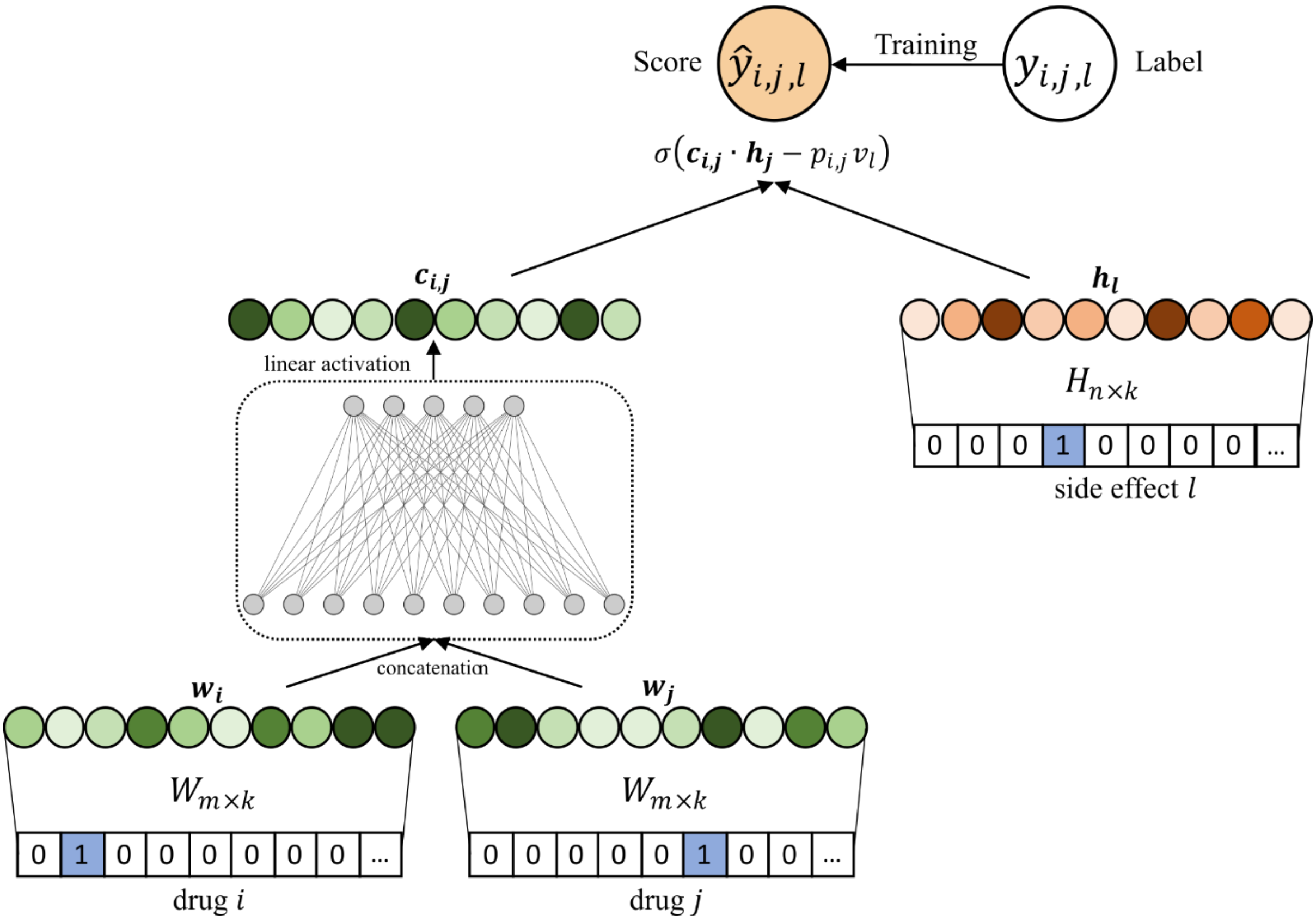

Fig A. *Overview of DCSE-linear.* The individual drug signatures are concatenated and fed to a single layer with linear activation to learn a signature for the combination. The final score is given by the dot product of the combination and side effect signature.

Table A. Performance comparison when combining the signatures linearly vs. nonlinearly.

Method	AUROC	AUPRC
**DCSE-linear**	78.4	77.5
**Decagon**	87.23	83.2
**DCSE**	91.71	87.24

Bias in DCSE

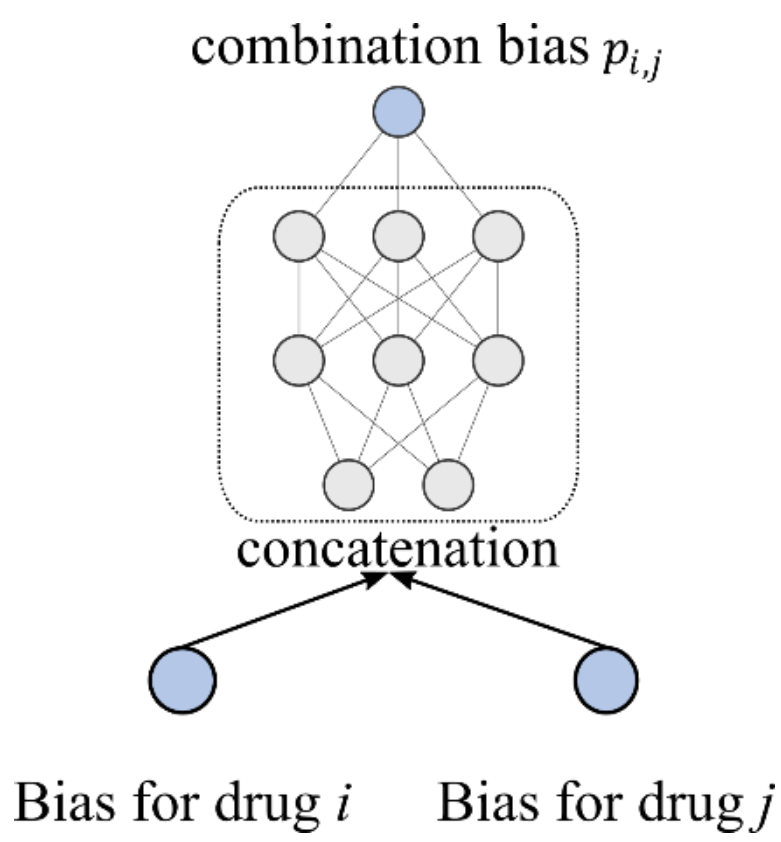

Fig B. *Architecture of the bias in the DCSE architecture*. The individual drug biases u_i and u_j are input to a small MLP that nonlinearly combines the biases to obtain a combination bias $p_{i,j}$.

What are the biases modelling?

To understand what the biases are capturing, we examined whether they model the popularity of drug pairs and side effects. Intuitively, the popularity of a drug pair in this context can be quantified by the number of side effects known to be associated with it. Similarly, side effect popularity can be measured by the number of drug pairs that have been reported to cause it. The more frequently a drug pair or side effect appears in the data, the more "popular" it can be considered. Formally, the popularity of a drug pair, denoted as $\mathrm{p}_{\mathrm{i,j}}^{\mathrm{pop}}$, is defined as the sum of row (i, j) in X, while the popularity of a side effect, $\mathrm{r}_{\mathrm{l}}^{\mathrm{pop}}$, is defined as the sum of column l in X:

$$p_{i,j}^{pop} = \sum_{l}^{n} X_{(i,j),l}, \quad r_{l}^{pop} = \sum_{(i,j)}^{m} X_{(i,j),l}.$$

If the biases are indeed capturing popularity, we would expect p and v to be negatively correlated with the vectors representing the popularity of drug pairs and side effects, $\overrightarrow{\mathrm{p}^{\mathrm{pop}}}$ and $\overrightarrow{\mathrm{r}^{\mathrm{pop}}}$, respectively. The assumption is that the model should generally assign higher scores to

more frequently observed drug pairs and side effects. Given that the bias terms are subtracted to the dot product of the signatures between the pair and the side effect before applying the sigmoid (see Equation 3 in *Materials and Methods*), higher values of p and v should lead to a larger subtraction, and thus, lower predicted scores. Conversely, lower values of p and v should result in higher predicted scores.

The Pearson correlation between $\overrightarrow{\mathrm{p}^{\mathrm{pop}}}$ and p is -0.816 (Fisher's two-sided exact test, $p < 2.68 \times 10^{-280}$), while for $\overrightarrow{\mathrm{r}^{\mathrm{pop}}}$ and v, the correlation is -0.680 ($p < 2.34 \times 10^{-179}$). These results confirm that the biases for drug pairs and side effects capture popularity. If these biases already model this aspect of the data, the signatures of the drugs and side effects should be able to focus on learning biologically meaningful interactions, enhancing the descriptive power of the learned representations.

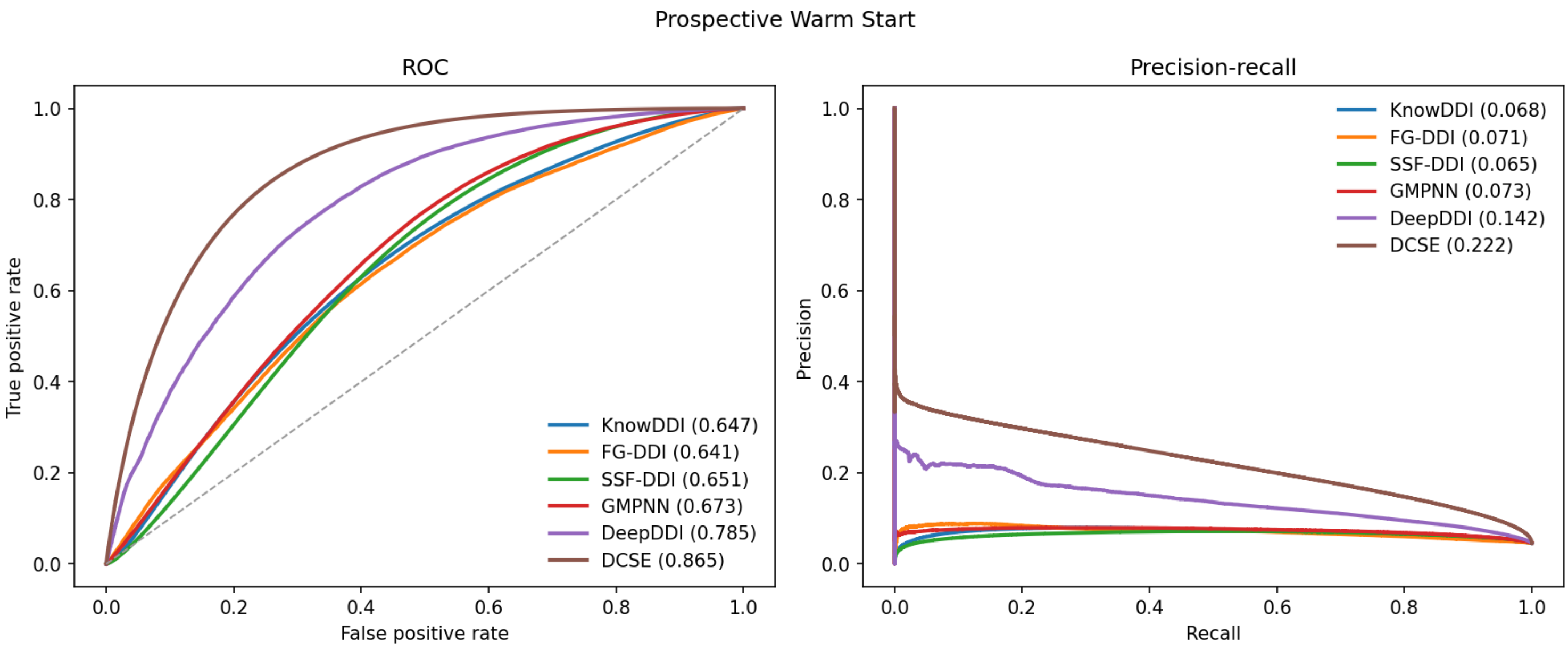

Fig C. *Prospective Warm Start ROC and PR curves.* DeepWalk and XGBoost are excluded from these curves because they are trained as separate binary classifiers for each side effect, whereas the ROC and PR curves shown here are computed from a single pooled set of predictions across all side effects.

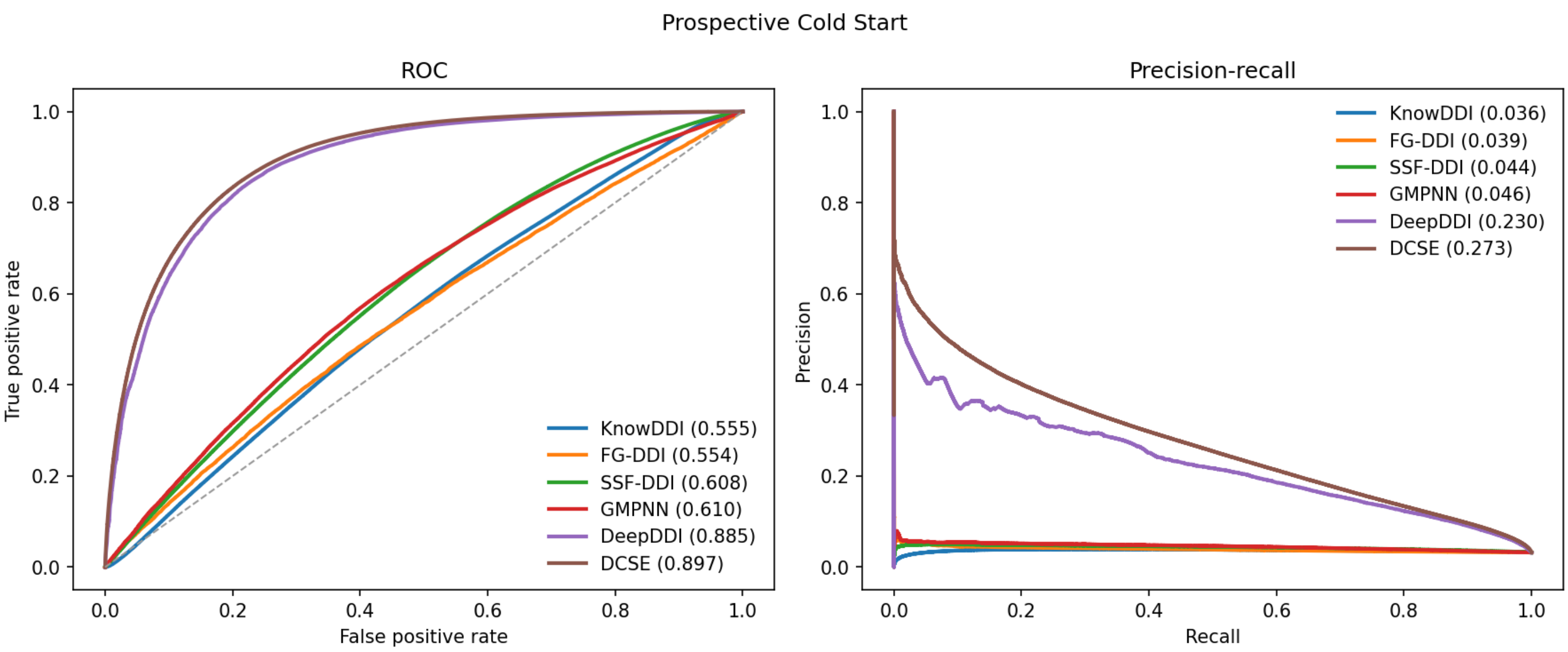

Fig D. *Prospective Cold Start ROC and PR curves.* DeepWalk and XGBoost are excluded from these curves because they are trained as separate binary classifiers for each side effect, whereas the ROC and PR curves shown here are computed from a single pooled set of predictions across all side effects.

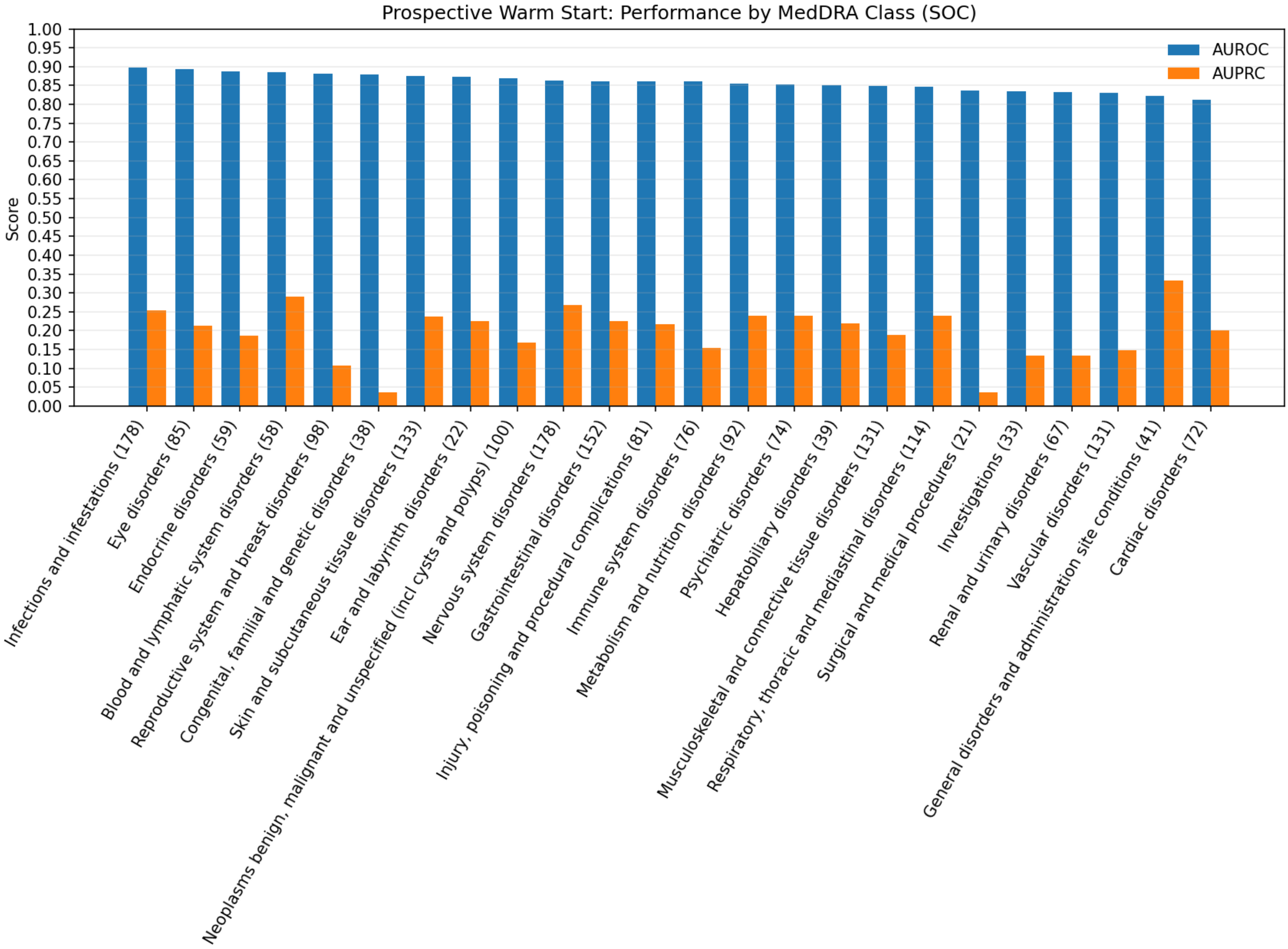

Fig E. *AUROC and AUPRC by MedDRA Class (SOC) in the Prospective Warm Start setting*. In parentheses we include the number of side effects within this class in the test set.

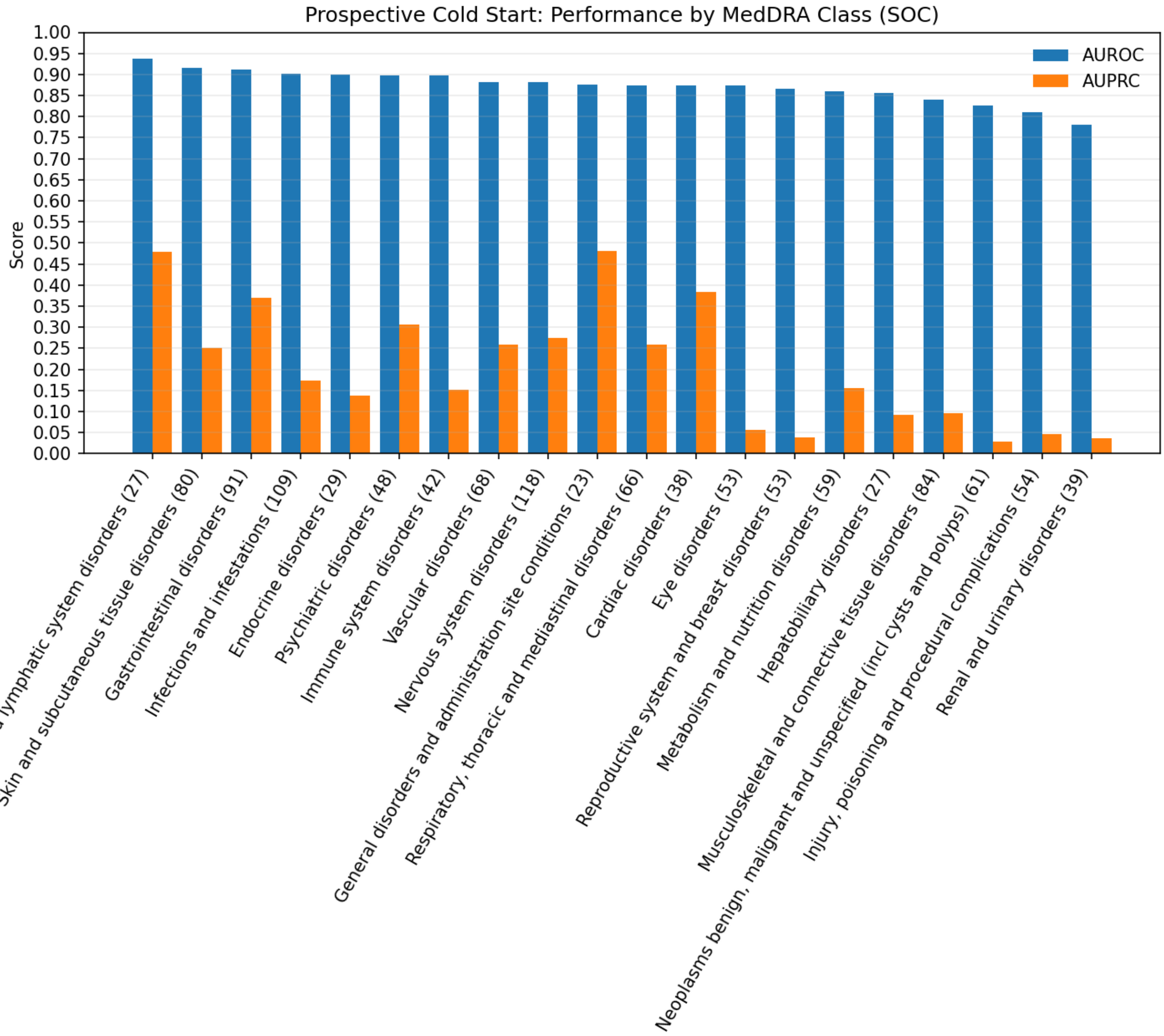

Fig F. *AUROC and AUPRC by MedDRA Class (SOC) in the Prospective Cold Start setting*. In parentheses we include the number of side effects within this class in the test set.
