# Supplementary material for: Robust prediction of drug combination side effects in realistic settings
Source: PLoS Comput Biol. 2026 Oct 5;22(10):e1013619. doi: 10.1371/journal.pcbi.1013619 (PMC13657403; doi:10.1371/journal.pcbi.1013619)
Supplement: Supplementary file 2 [file pcbi.1013619.s002.pdf]

Interaction evidence for the top DCSE cold-start predictions

Unique drug pairs appearing in the top 50 DCSE predictions for the cold-start prospective set.

Drugs.com reports pair-level clinical interaction warnings, with severity labels and adverse-effect descriptions derived from Cerner Multum interaction content.

DrugBank reports curated interaction descriptions, typically phrased as changes in pharmacological activity, exposure, efficacy, toxicity, or adverse-effect risk.

We use these resources as external evidence to assess whether the highest-ranking DCSE cold-start predictions correspond to drug pairs with independently reported interaction signals.

Drug pair	Drugs.com information	DrugBank information
fluoxetine + naratriptan	Major interaction. Drugs.com reports increased risk of serotonin syndrome when fluoxetine is used with naratriptan. Drugs.com	Increased risk or severity of serotonin syndrome.
amoxicillin + nefazodone	No Drugs.com interaction warning identified.	Increased risk or severity of liver damage.
baclofen + nefazodone	Moderate interaction. Drugs.com reports increased dizziness, drowsiness, confusion, difficulty concentrating, and impaired thinking, judgment, and motor coordination. Drugs.com	Baclofen may increase the CNS depressant activities of nefazodone.
anastrozole + granisetron	No Drugs.com interaction warning identified.	Increased risk or severity of liver damage.
amitriptyline + naratriptan	Major interaction. Drugs.com reports increased risk of serotonin syndrome when amitriptyline is used with naratriptan. Drugs.com	Increased risk or severity of liver damage.
azithromycin + progesterone	No Drugs.com interaction warning identified.	Increased risk or severity of liver damage.
azithromycin + phentermine	No Drugs.com interaction warning identified.	No DrugBank interaction provided.
baclofen + oxaprozin	No Drugs.com interaction warning identified.	Oxaprozin may decrease the excretion rate of baclofen, potentially increasing baclofen serum level.
baclofen + meperidine	Major interaction. Drugs.com reports risk of serious CNS and respiratory depression, such as profound sedation, respiratory distress, coma, and death. Drugs.com	Baclofen may increase the CNS depressant activities of meperidine.
amoxicillin + oxaprozin	No Drugs.com interaction warning identified.	Increased risk or severity of liver damage.
gemcitabine + tramadol	Drugs.com reports no interactions found between Gemzar (gemcitabine) and tramadol. Drugs.com	Increased risk or severity of liver damage.
flutamide + thalidomide	Moderate interaction. Drugs.com reports that thalidomide can slow heart rate or cause irregular rhythm, and combination with flutamide may increase that risk. Drugs.com	Increased risk or severity of liver damage.
granisetron + tamsulosin	No Drugs.com interaction warning identified.	Increased risk or severity of liver damage.
azithromycin + gemcitabine	No Drugs.com interaction warning identified.	Increased risk or severity of liver damage.

Continued on next page

Drug pair	Drugs.com information	DrugBank information
amitriptyline + imipramine	Moderate interaction. Drugs.com reports increased side effects such as drowsiness, blurred vision, dry mouth, constipation, irregular heartbeat, confusion, and memory problems; it also flags therapeutic duplication for tricyclic/tetracyclic antidepressants. Drugs.com	Imipramine may decrease the metabolism of amitriptyline.
naratriptan + tizanidine	Moderate interaction. Drugs.com reports increased dizziness, drowsiness, confusion, difficulty concentrating, impaired thinking/judgment/motor coordination, and additive blood-pressure-lowering effects. Drugs.com	Increased risk or severity of liver damage.
ampicillin + granisetron	No Drugs.com interaction warning identified.	Increased risk or severity of liver damage.
benazepril + thalidomide	No Drugs.com interaction warning identified.	Increased risk or severity of liver damage.
benazepril + gemcitabine	No Drugs.com interaction warning identified.	Increased risk or severity of liver damage.
azithromycin + imipramine	Moderate interaction. Drugs.com reports increased risk of a serious, potentially life-threatening irregular heart rhythm. Drugs.com	Increased risk or severity of liver damage.

Summary. The top 50 DCSE cold-start predictions contain 20 unique drug pairs. Drugs.com reports interaction warnings for 8 of 20 unique pairs, and DrugBank information was provided for 19 of 20 unique pairs. This provides encouraging external support for the applicability of DCSE to previously uncharacterized drug combinations in prospective settings.
